# Alpha Particle Effect on Multi-Nanosheet Tunneling Field-Effect Transistor at 3-nm Technology Node

**DOI:** 10.3390/mi10120847

**Published:** 2019-12-04

**Authors:** Jungmin Hong, Jaewoong Park, Jeawon Lee, Jeonghun Ham, Kiron Park, Jongwook Jeon

**Affiliations:** Department of Electrical and Electronics Engineering, Konkuk University, Seoul 05029, Korea; hjmin0208@naver.com (J.H.); qkrwodnd789@naver.com (J.P.); leejw4417@naver.com (J.L.); jhham5969@naver.com (J.H.);

**Keywords:** reliability, radiation effect, alpha particle, nanosheet tunneling field-effect transistor (FET)

## Abstract

The radiation effects on a multi-nanosheet tunneling-based field effect transistor (NS-TFET) were investigated for a 3-nm technology node using a three-dimensional (3D) technology computer-aided design (TCAD) simulator. An alpha particle was injected into a field effect transistor (FET), which resulted in a drain current fluctuation and caused the integrated circuit to malfunction as the result of a soft-error-rate (SER) issue. It was subsequently observed that radiation effects on NS-TFET were completely different from a conventional drift-diffusion (DD)-based FET. Unlike a conventional DD-based FET, when an alpha particle enters the source and channel areas in the current scenario, a larger drain current fluctuation occurs due to a tunneling mechanism between the source and the channel, and this has a significant effect on the drain current. In addition, as the temperature increases, the radiation effect increases as a result of a decrease in silicon bandgap energy and a resultant increase in band-to-band generation. Finally, the radiation effect was analyzed according to the energy of the alpha particle. These results can provide a guideline by which to design a robust integrated circuit for radiation that is totally different from the conventional DD-FET approach.

## 1. Introduction

As semiconductor technology continues to scale down, a 5-nm technology node has been produced that is considered to be the most significant three-dimensional (3D) fin field-effect transistor (FinFET) architecture utilized since the 22-nm technology node [1,2,3,4,5]. Recently, 3D gate-all-around semiconductor devices, such as multi-nanowire and multi-nanosheet devices, have been attracting attention [6,7,8]. Carrier transport from the planar MOS field-effect transistor (MOSFET) to the 3D FinFET is based on the drift-diffusion (DD) transport mechanism. To overcome the limitations of conventional DD carrier transport-based FET (DD-FET), a tunneling carrier transport-based FET (T-FET) has been considered as a next generation semiconductor device due to its advantages of an improved short-channel effect, subthreshold swing (SS) and off-state current value [9,10,11,12,13]. Usually T-FET has a different source and drain-doping scheme than the conventional DD-FET to modulate the generated tunneling effect. The flow of electrons through the thin energy bandgap between the source and channel regions increases the on/off current ratio and behaves as a highly efficient logic switch, reducing the leakage current. In addition, the improved SS characteristic of the T-FET can lower the off-current value of the device, which lowers the operating voltage and enables power consumption reduction, a very critical problem in scaling down the device.

Meanwhile, reliability issues due to the effects of radiation have been considered to be very important in semiconductor device development for a long time, as they can cause sudden abnormal semiconductor operations. As semiconductor devices become smaller, the probability of exposure to radiation decreases, but the signal-to-noise margin of the device decreases simultaneously, meaning that the effects of radiation remains an important reliability analysis factor [14,15,16]. When high-energy particles are injected into the semiconductor device, they releases energy inside the substrate, and immediately producing electrons and holes (electron hole pair (EHP) generation). The generated electrons are collected by the electric field due to the applied drain voltage, and suddenly appear in the current flowing through the device. This is called a “soft error”, because it does not cause direct physical damage to the element, and does not persist in the error state, which causes misbehavior of the integrated circuit [17,18,19]. Particles that cause soft errors typically include α-particles from materials used in processes or packaging and cosmic radiation such as protons, neutrons, muons and many other secondary particles and heavy ions. These inject the semiconductor device with various energies, with the energy range varies depending on the particle type. α-particles are mainly emitted from gold and lead, which are used in the packaging of the device and have an energy range of 1–9 MeV. Many research papers on the effects of particle radiation on semiconductor devices has been presented in the past, and recently the results of particle radiation on 5-nm technology nodes using a 3D vertical FET based on conventional drift-diffusion carrier transport have been published [20,21,22,23,24]. However, there are no analyses of the effects of particle radiation on multi-nanosheet tunneling-based field effect transistors (NS-TFETs) based on the tunneling mechanism in a 3D multi-nanosheet structure that are highly applicable to the 3-nm technology node. In this work, the incident location, incident energy, and various operating temperature conditions were analyzed for alpha particles in a NS-TFET structure with a 3-nm technology node dimension. Three-dimensional technology computer-aided design (TCAD) simulation software, which supports the alpha particle characteristics in silicon, was used. The effect of particle radiation on NS-TFET was significantly different from that of other conventional DD-FETs, and an analysis of the results was carried out. These results suggest that the NS-TFET requires a completely different design approach for optimizing the drain area in order to be less affected by radiation when designing an integrated circuit using the conventional DD-FET [25,26,27].

## 2. Three-Dimensional (3D) Device and Methodology

**Figure 1:**
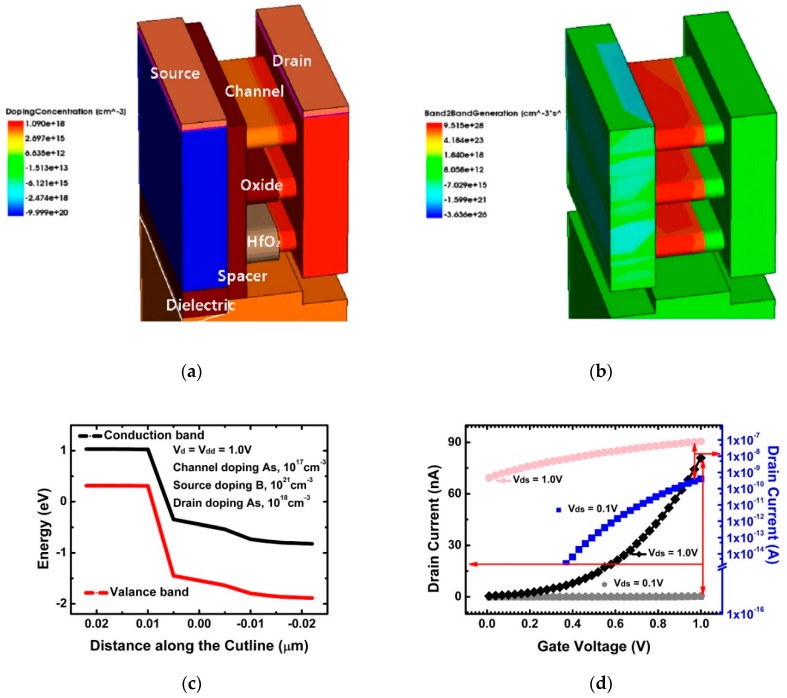
(**a**) Structure and doping concentration of the 3-nm multi-nanosheet, (**b**) band-to-band tunneling generation, (**c**) energy band diagram and (**d**) *I*_ds_-*V*_gs_ transfer curve.

Figure 1 shows the 3D structure of a 3-nm node NS-TFET in Synopsys Sentaurus TCAD software (Synopsys, Mountain View, CA, USA) that was used to simulate an NS-TFET providing model incidents on a silicon semiconductor [28]. Design specifications were determined according to the international technology roadmap for 2015 [29]. Gate length (*L*_g_) was set to 10 nm. Channel width (*T*_w_) and channel thickness (*T*_ch_) were 16 and 5 nm, respectively. The contact distance from the middle of the source to that of the drain was 23.5 nm. Effective oxide thickness (EOT) was set to 0.68 nm (*T*_ox_ = 0.43 nm, *T*_hk_ = 1.4 nm), ensuring gate controllability. Doping at the source, drain and channel were set to 10^21^ cm^−3^ boron, 10^18^ cm^−3^ arsenic, and 10^17^ cm^−3^ arsenic, respectively. Note that while optimizing the doping concentration can further improve a device’s T-FET performance, there is no significant change in the behavior of the alpha particles when compared to conventional DD-FET. Contact resistance was set to 3 × 10^−9^ Ohm/cm^2^ considering latest process technology. Threshold voltage was controlled by the gate work function of the gate electrode material.

For implementation of the alpha-particle behavior in the 3-nm-node NS-TFET in Synopsys’ TCAD, a drift-diffusion approximation was used for the carrier transport. In addition to a non-local tunneling model and alpha particle incidence dimension, the current-voltage measured data of fabricated 14-nm bulk FinFET was used to calibrate the TCAD model. First, the 3D structure of the mobility degradation was plotted using a Lombardi method along with a thin-layer mobility model, as outlined in a previous work [25]. Next, the calibrated model parameters were applied to our TCAD simulation for the 3-nm NS-TFET.

In order to observe the alpha particle behavior incident on the NS-TFET, a test was performed by changing the points of incidence, angles of incidence, and temperature. The points of incidence were the source, drain, and channel regions; the angles of incidence was 30–90°; and the alpha particle energy was designated as 1–10 MeV. To take into account the carrier temperature, the hydro-dynamic model was turned on in the physics section of the TCAD. In addition, a non-local band-to-band tunneling model was used to consider the tunneling current dependency on the band edge profile along the entire path between the points connected by tunneling [30]. The device dimension and simulation conditions are listed in Table 1, and the doping concentration and band-to-band tunneling generation are described in Figure 1a,b, respectively. The following graph, Figure 1c, is a band diagram to determine if a device is acting as a tunneling FET. The valance and conduction bands in Figure 1c are bent to allow tunneling between bands between the source and channel [31,32,33,34]. In addition, Figure 1d shows the characteristics of *I*_ds_-*V*_gs_. When the current values of *V*_ds_ = 1.0 V and *V*_ds_ = 0.1 V are compared with *V*_gs_ = 1.0 V, the difference of the current value between the two different *V*_ds_ is 80.5 nA.

## 3. Results and Discussion

**Figure 2:**
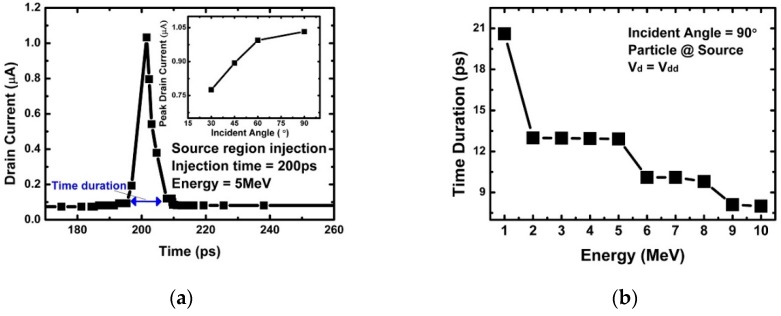
(**a**) Transient drain current of the multi-nanosheet tunneling-based field-effect transistor (NS-TFET) when an alpha particle is injected into the source region at *t* = 200 ps; (**b**) time duration with different particle energy injections at the source with an incident angle of 90°.

**Figure 3:**
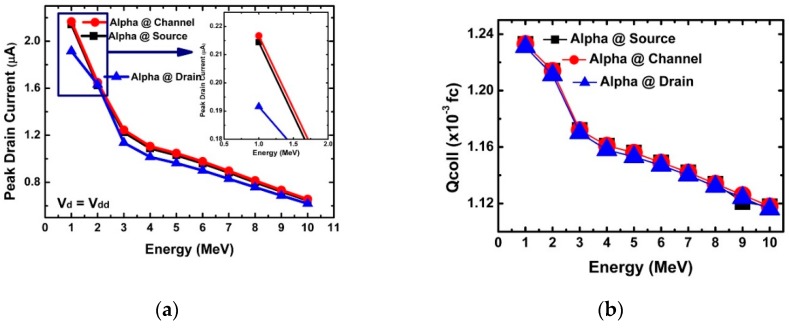
(**a**) Peak drain current with particle energy in different regions; (**b**) collected charges of different particle energy injections at the source, channel, and drain with an incident angle of 90°.

Figure 2a shows the variation of the drain current over time when an alpha particle enters the NS-TFET. This experiment is the result of injecting an alpha particle into the center of the source region with 5 MeV energy at *t* = 200 ps when the NS-TFET channel is in inversion and operating in the saturation region. When the alpha particle was injected, electron hole pair (EHP) generation occurred along the incident path, and the generated EHP contributed to the drain current component according to the electric field profile inside the NS-TFET. The drain current fluctuations increased during the short time period just after the alpha particle was injected, then returned to a steady state due to EHP recombination. At that time, not all EHPs generated by the alpha particles contributed to the drain current, and only the surviving carriers contributed to the current without recombination before the generated EHP reached the drain region. It was expected that the magnitude of the peak drain current due to the alpha particles would be much larger than the steady-state current (see Figure 2) and cause circuit malfunction. The inset in Figure 2 shows the peak drain current according to the alpha particle incidence angle. The closer the incidence angle was to the vertical, the larger the peak drain current became. Figure 2a marks the time duration. When the incidence position was applied to the source, channel, and drain, the time duration value according to the incident energy was almost take the same, as shown in Figure 2b. It can be observed in Figure 2b that the time duration also decreased as the incident energy increased. The graph in Figure 2b depicts how the time duration value decreased as the energy increased which is similar to Figure 3a that depicts how the peak drain current decreased with the incident energy. Therefore, there is a correlation between the amount of EHP generated by alpha particle incidence, which affects the peak drain current according to alpha particle incidence [35,36,37,38,39].

**Figure 4:**
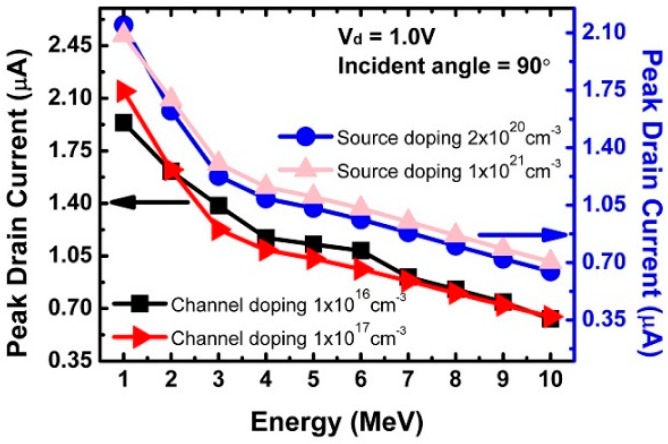
Peak drain current and particle energy in the source and channel regions by different source and channel doping concentrations.

Figure 4 shows peak drain current with different doping concentration when the source was fixed to 1 × 10^20^ cm^−3^ and the doping concentration of the channel was set to 1 × 10^16^ and 1 × 10^17^ cm^−3^. When the energy was its lowest, the device with the higher doping concentration showed a higher peak drain current. The doping concentration of the source was doped into 2 × 10^20^ and 1 × 10^21^ cm^−3^, and the channel was fixed at 1 × 10^17^ cm^−3^. In the different source doping concentration case, the peak drain current was high at the low doping concentration when the incident energy was 1 MeV. However, it was confirmed that both trends reversed as the incident energy surpassed 2 MeV.

Figure 3a,b shows the variation of the peak drain current and collected charge according to the incidence point and incident energy of the alpha particles. The collected charge can be obtained by integrating the current variation over time. Figure 3a shows that when the alpha particles were injected into the channel and source regions, they had a larger peak drain current than when they were injected into the drain, which was the opposite of a conventional DD-FET. For a DD-FET, the influence of the alpha particle injection into the drain is greater than at the source and channel regions [31]. It can be interpreted that the variation of EHP concentration in the channel and source regions has a big influence on the T-FET current because tunneling between the channel and source is the most important factor in determining the drain current in T-FETs.

**Figure 5:**
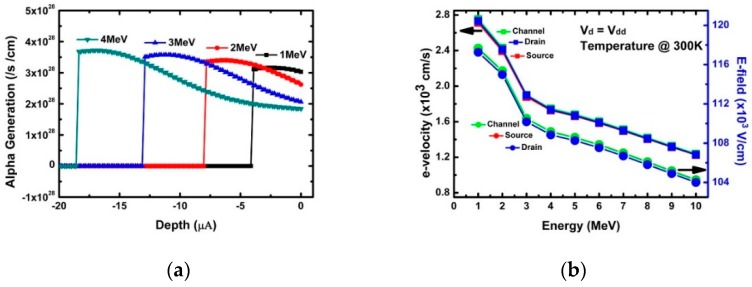
(**a**) Generation distribution with energy of particles; (**b**) electric field and electron velocity at the top of the drain bulk with the different particle energies.

In addition, Figure 3 shows that the peak drain current and collected charge decreased as the energy of the incident alpha particle increased. This can be explained by the EHP generation trend according to particle energy, as shown in Figure 5a. It can be seen that as the particle energy increased, the area where peak EHP was generated became further away from the incident point. Therefore, it can be concluded that EHPs generated by high particle energy cannot contribute to drain current fluctuations because EHPs generated by particles recombine before reaching the drain region. Drain current fluctuations are affected by electric field and carrier velocity fluctuations in addition to the EHP density generated by alpha particles [25]. Figure 5b shows electron velocity (e-velocity) and an electric-field (E-field) variations inside the NS-TFET according to particle energy. As the particle energy increased, both the e-velocity and E-field decreased, which is different from the vertical-FET (V-FET) result [25]. In V-FET, the higher particle energy increases both values. However, the results of this work are the same as for the device where the source and drain are located in the horizontal direction, as with common FinFETs.

**Figure 6:**
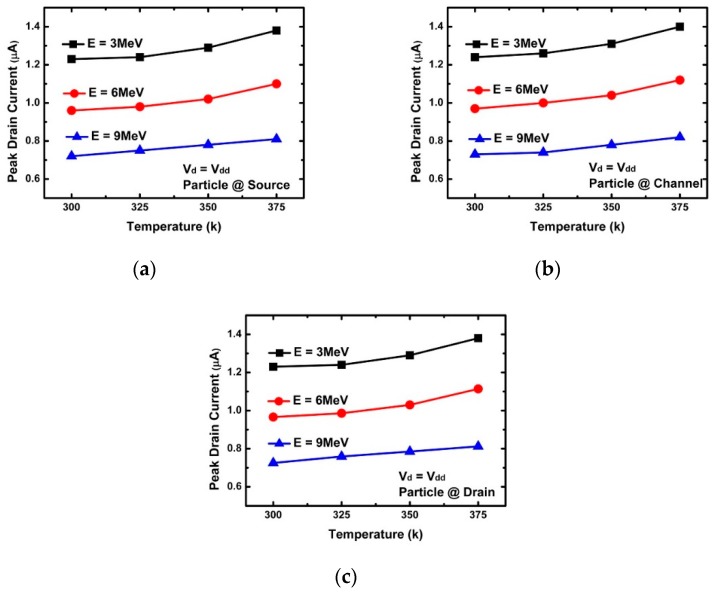
Peak drain current with three different alpha particle energy injections (3, 6, and 9 MeV) at the (**a**) source (**b**) channel, and (**c**) drain.

**Figure 7:**
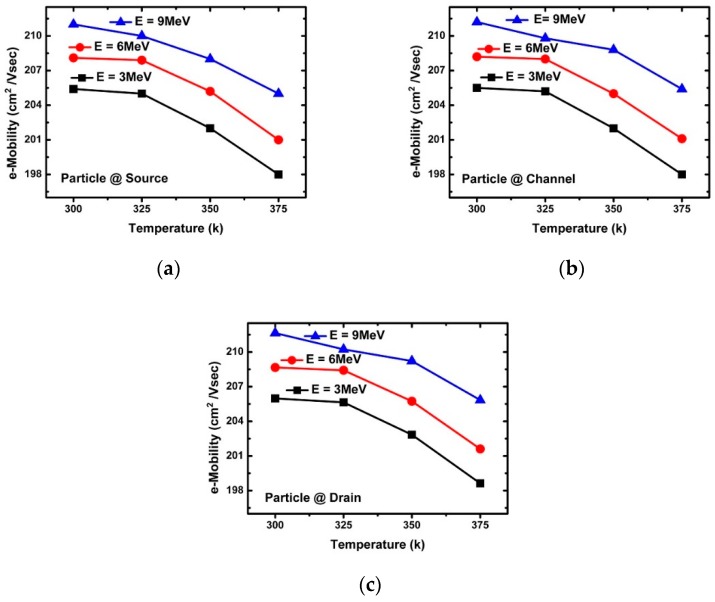
E-mobility with three different alpha particle energy injections (3, 6, and 9 MeV) at the (**a**) source, (**b**) channel, and (**c**) drain.

**Figure 8:**
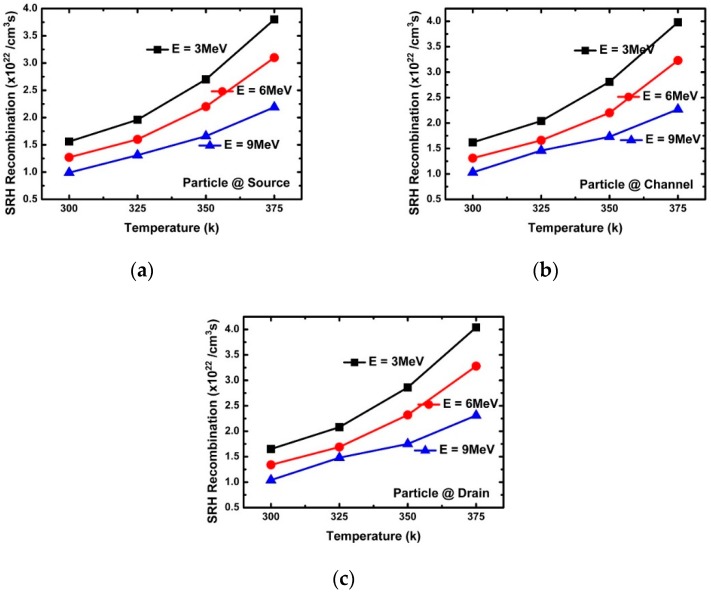
Shockley–Read–Hall (SRH) recombination with three different alpha particle energy injections (3, 6, and 9 MeV) at the (**a**) source, (**b**) channel, and (**c**) drain.

**Figure 9:**
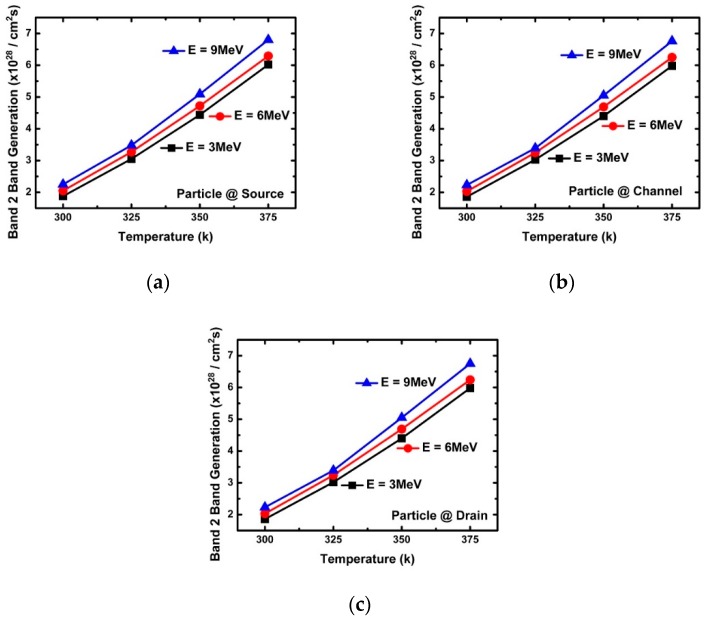
Band-to-band tunneling generation with three different alpha particle energy injections (3, 6, and 9 MeV) at the (**a**) source, (**b**) channel, and (**c**) drain.

**Figure 10:**
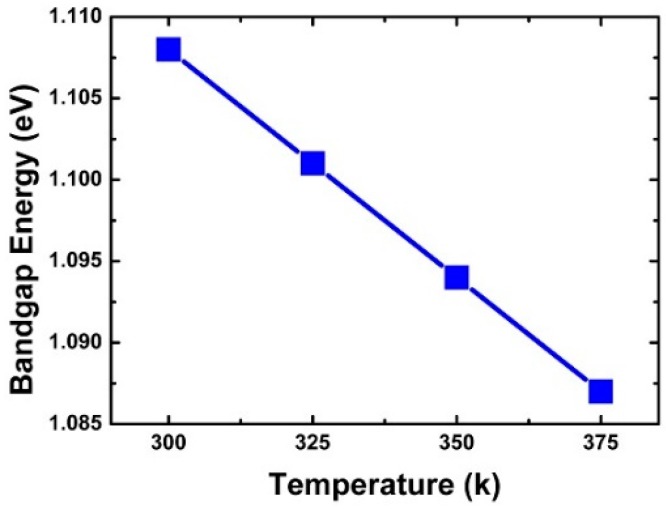
Silicon energy bandgap with different temperatures.

Figure 3a confirms the change of the peak drain current according to energy. The decrease in peak drain current with increasing incident energy was found to be greatly affected by the penetration depth, e-velocity, and E-field of the incident alpha particles. Figure 5, Figure 6, Figure 7, Figure 8 and Figure 9 reflect a change in temperature. Figure 5, Figure 6, Figure 7, Figure 8 and Figure 9 show that all the electrical behaviors of various injected particle energies had the same trend for three incident energies. Figure 6 shows the results of alpha particle effects at various ambient temperature conditions. In the experiment of ambient temperature variation, the alpha particle effect was observed by varying the point of particle incidence and incident particle energy, the channel of the NS-TFET was strongly inverted, and the NS-TFET operated in the saturation region. It can be observed in Figure 6 that the peak drain current caused by the alpha particles increased when the ambient temperature increased. This result is the opposite of a previous work regarding FinFET and V-FET [25]. To analyze the results, we plotted electron mobility (e-mobility) and Shockley–Read–Hall (SRH) recombination according to ambient temperature, as shown in Figure 7 and Figure 8, respectively. As the ambient temperature increased, e-mobility decreased and SRH recombination increased. This is the same result as FinFET and V-FET [25]. Note that in this work, the used TCAD mobility model and parameters were taken from [25]. Although the temperature dependence of NS-TFET’s e-mobility and SRH recombination was the same as that of FinFET and V-FET, bandgap energy was analyzed to identify why the temperature dependence of the alpha particle of the NS-TFET was different from that of FinFET and V-FET. As the ambient temperature increased, the bandgap energy decreases, as shown in Figure 10. Furthermore, as the bandgap energy decreased, band-to-band tunneling between the source and channel increased, as shown in Figure 9. Therefore, the increase in peak drain current according to alpha particles and temperature can be explained by an increase in the tunneling current in NS-TFET resulting from the bandgap narrowing effect.

## 4. Conclusions

In this work, we analyzed the radiation effect caused by alpha particles in multi-nanosheet tunneling-TFET (NS-TFET) in a 3-nm technology node using 3D TCAD simulation. Due to the stacked multi-nanosheet structure and carrier transport of the tunneling mechanism in a lateral direction, the drain current variation was different from that of FinFET and V-FET when alpha particles are injected into the NS-TFET. First, when the alpha particle is injected into the channel and source regions, the effects of radiation are greater than when they are injected into the drain region. Second, the peak drain current fluctuation due to alpha particles decreases monotonically according to the incident energy. Third, as the ambient temperature increases, the peak drain current fluctuation increases monotonically due to the alpha particles. As a result of analyzing various physical characteristics through 3D TCAD, it has been observed that these characteristics are unique radiation characteristics shown by the structure and carrier transport of NS-TFET. Therefore, it can be seen that a design method different from the previous DD-based FETs is required for the development of integrated circuits that are resistant to radiation using NS-TFETs.

## Figures and Tables

**Table 1:** Used parameters for device geometry and alpha particle simulation, which correspond to the international technology roadmap for semiconductors (ITRS)-based 3-nm technology node scenario.

Device Parameters	Value	Device Parameters	Value
Gate length, *L*_g_ (nm)	10	Ambient temperature (K)	300–375
Channel width, *T*_w_ (nm)	16	Alpha particle energy (MeV)	1–10
Channel thickness, *T*_ch_ (nm)	5	Incident angle (°)	30–90
Contact gate pitch, CGP (nm)	23.5	Source doping (cm^−3^)	B, 10^21^
Effective oxide thickness (nm)	0.68	Drain doping (cm^−3^)	As, 10^18^
Contact resistance (Ω-cm^−3^)	3 × 10^−9^	Channel doping (cm^−3^)	As, 10^17^
Gate voltage (*V*_g_) (V)	0–1.0	Drain voltage (*V*_d_) (V)	0.1, 1.0
